# Performance of DeepSeek-R1, ChatGPT (GPT-o3-mini), and Gemini 2.0 Flash on German Medical Multiple-Choice Questions: Comparative Evaluation

**DOI:** 10.2196/77357

**Published:** 2025-12-18

**Authors:** Annika Meyer, Yassin Karay, Andrea U Steinbicker, Thomas Streichert, Remco Overbeek

**Affiliations:** 1 Department of Anesthesiology and Operative Intensive Care Faculty of Medicine and University Hospital University Hospital Cologne Cologne Germany; 2 Dean’s Office for Student Affairs Faculty of Medicine University Hospital Cologne Cologne Germany; 3 Institute for Clinical Chemistry Faculty of Medicine and University Hospital University Hospital Cologne Cologne Germany

**Keywords:** ChatGPT, Gemini, DeepSeek, large language model, chatbots, artificial intelligence

## Abstract

**Background:**

Despite the transformative potential of artificial intelligence (AI)–based chatbots in medicine, their implementation is hindered by data privacy and security concerns. DeepSeek offers a conceivable solution through its capability for local offline operations. However, as of 2025, it remains unclear whether DeepSeek can achieve an accuracy comparable to that of conventional, cloud-based AI chatbots.

**Objective:**

This study aims to evaluate whether DeepSeek, an AI-based chatbot capable of offline operation, achieves answer accuracy on medical multiple-choice questions (MCQs) comparable to that of leading chatbots (ie, ChatGPT and Gemini) on German medical MCQs, thereby assessing its potential as a privacy-preserving alternative for clinical use.

**Methods:**

A total of 200 interdisciplinary MCQs from the German Progress Test Medicine were administered to ChatGPT (GPT-o3-mini), DeepSeek (DeepSeek-R1), and Gemini (Gemini 2.0 Flash). Accuracy was defined as the proportion of correctly solved questions. Overall differences among the 3 models were tested with the Cochran Q test, while pairwise comparisons were conducted using the McNemar test. Subgroup analyses were performed by medical domain (Fisher exact test) and question length (Wilcoxon rank-sum test). An a priori power analysis indicated a minimum sample size of 195 questions.

**Results:**

All 3 chatbots surpassed the conventional passing threshold of 60%, with accuracies of 96% (192/200) for DeepSeek, 94% (188/200) for Gemini, and 92.5% (185/200) for ChatGPT. The overall difference among models was not statistically significant (*P*=.10) nor were pairwise comparisons. However, incorrect responses were significantly associated with longer question length for DeepSeek (*P*=.049) and ChatGPT (*P*=.04) but not for Gemini. No significant differences in performance were observed across clinical versus preclinical domains or medical specialties (all *P*>.05).

**Conclusions:**

Overall, DeepSeek demonstrates outstanding performance on German medical MCQs comparable to the widely used chatbots ChatGPT and Gemini. Similar to ChatGPT, DeepSeek’s performance declined with increasing question length, highlighting verbosity as a persistent challenge for large language models. While DeepSeek’s offline capability and lower operational costs are advantageous, its safe and reliable application in clinical contexts requires further investigation.

## Introduction

In recent years, artificial intelligence (AI) has experienced a remarkable surge in development and adoption [1]. Although applications in medicine are not entirely new, with substantial investments in health care–related AI already initiated nearly a decade ago, the capabilities of AI have advanced considerably [1,2]. These developments offer opportunities for research, automation of routine tasks, and diagnostic support while simultaneously raising persistent ethical, governance, and regulatory challenges [1,3].

A major catalyst for the rapid uptake of AI has been the publication of AI-based chatbots on the World Wide Web, which have substantially lowered barriers related to the usability and accessibility of sophisticated AI systems [4]. Their applications in medicine and research are increasingly under investigation. For example, ChatGPT (OpenAI), introduced in 2022, demonstrates strong performance in medical state examinations, can generate medical reports and radiology documentation, and supports medical programming [5-7]. In addition, these chatbots exhibit considerable linguistic fluency and empathy in response to patients’ inquiries, although answer quality can vary across medical specialties [8,9].

Despite their promising capabilities, relevant concerns regarding data privacy and security impede the clinical adoption of online AI-based chatbots in hospitals [4]. One proposed solution is the recently introduced offline-capable chatbot, DeepSeek (Hangzhou DeepSeek Artificial Intelligence Basic Technology Research Co, Ltd) [10]. Notably, initial analyses already suggest that DeepSeek excels in various benchmarks, such as mathematics-based assessments, surpassing previous chatbot iterations [11,12]. Developed by a small team of computer scientists in 2025 [13,14], DeepSeek gained immediate prominence due to its efficient computational design that requires fewer graphical processing units. Operational costs are thereby reduced, and user fees and the carbon footprint are lowered [12-15]. Moreover, its partially open-source nature might further encourage ongoing innovation of AI [10,13,14].

However, empirical evidence on the accuracy of answers on medical multiple-choice questions (MCQs) compared with established chatbots remains scarce, underscoring the need for systematic evaluation [10,12]. Therefore, this study aimed to systematically evaluate DeepSeek’s performance in applying medical knowledge by comparing it with ChatGPT and Gemini (Google DeepMind) on 200 MCQs from the German Progress Test Medicine (PTM).

## Methods

### Berlin Progress Test

The PTM used in this study is a knowledge test designed at the Charité in Berlin for students of human medicine. It is taken by approximately 12,000 students from 19 universities in Germany, Austria, and Switzerland through their medical studies. The PTM consists of 200 interdisciplinary MCQs at the graduate level and is designed to provide students with objective feedback on their personal growth in knowledge over the course of their studies. The questions cover a broad spectrum of domains, including internal medicine, surgery, pediatrics, obstetrics and gynecology, psychiatry, anesthesiology, radiology, laboratory medicine, and the basic sciences (eg, anatomy, physiology, and biochemistry), ensuring balanced representation of both clinical and preclinical content [16]. By covering both clinical and preclinical domains, the test provides a balanced and integrated assessment of medical knowledge, which was the rationale for its selection in this study. Moreover, the test results and, in particular, the knowledge gained per semester have proven to be suitable criteria for predicting academic success with regard to the German state examinations [17]. For this study, we used the 51st PTM, published in October 2024, which had a mean discrimination index of 0.45 and a Cronbach α of 0.98 [16]. Each question was categorized by a physician according to the subject area and study phase (eg, clinical phase and preclinical phase).

### Data Collection

To address our research question, we evaluated the performance of 3 chatbots, ChatGPT (GPT-o3-mini), DeepSeek (DeepSeek-R1), and Gemini (Gemini 2.0 Flash), using 200 MCQs from the PTM 51 published in October 2024 between February 21, 2025, and March 4, 2025. These chatbots were selected because they represent 3 leading approaches to large language models in medicine: ChatGPT as the widely used benchmark, Gemini as a major proprietary competitor, and DeepSeek as a novel offline-capable alternative with potential privacy advantages. The specific versions were chosen because they were the most recent publicly accessible releases and free of cost at the time of data collection, reflecting the default user-facing performance available in February 2025.

All questions were used with formal permission, presented in German, and included without modification or exclusion. The number of the included 200 questions was determined based on a sample size calculation for the McNemar test, assuming a Cohen *d* of 0.28 and a statistical power of 80%, yielding a required sample size of 195. In accordance with the findings of Alfertshofer et al [18], the word count of each question was subsequently determined.

Each unaltered question was entered into the publicly accessible default web-based interface of each chatbot without supplementary prompting or user-directed modifications. Browsing and integrated tools were left enabled, reflecting the standard user-facing functionality of each system. To avoid memory effects or response contamination, each question was submitted in a separate, newly initiated chat session. On occasions where the chatbot failed to generate a response initially, the query was reinitiated.

In addition to the quantitative analyses, 2 physicians independently conducted an exploratory qualitative review of discrepancies between the officially defined correct answers and the responses generated by the chatbots. Potential reasons for discrepancies were derived through independent review and subsequent discussion, without the application of a predefined coding framework, aimed at providing illustrative examples of chatbot limitations.

### Statistical Analysis

Statistical analyses were performed using “R” (version 2025.09.1+401: R Foundation for Statistical Computing) [19]. Sample size calculations were performed with the *pwr* package [20]. Data wrangling and analysis were carried out using *rio* [21], *tidyverse* [22], *gtsummary* [23], *rstatix* [24], *labelled* [25], and *fastDummies* [26], while data visualization was accomplished using the *tidyverse* [22], *RColorBrewer* [27], and *cowplot* packages [28]. ChatGPT was used to facilitate statistical programming, while all AI-generated content was critically reviewed by a human. Categorical variables were summarized by absolute and relative frequencies, whereas continuous variables were described using medians and IQRs. Normality was assessed using the Shapiro-Wilk test. Differences in chatbot performance were evaluated using the McNemar test for paired categorical data with Bonferroni correction and the Fisher exact test for unpaired categorical data, while the Wilcoxon rank-sum test was applied for continuous data. A binomial test was applied to test the performance of the chatbots against the threshold of 60%. A *P* value of <.05 was considered statistically significant (Multimedia Appendices 1 and 2).

### Ethical Considerations

As this study was limited to medical state examination questions and publicly available results, no research involving human participants was conducted. In accordance with the guidelines of the ethics committee of the University of Cologne, ethics approval was therefore not required [29].

## Results

### Question Characteristics

A total of 200 original medical progress test questions were used to evaluate the 3 chatbots, with an average length of 55 (IQR 40-74) words. Most (177/200, 88.5%) questions focused on clinical knowledge, with nearly one-quarter (47/200, 23.5%) specifically addressing internal medicine (Table 1).

**Table 1:** Comparison of DeepSeek, Gemini, and ChatGPT in the Progress Test Medicine.

Characteristic		All questions (N=200)	DeepSeek					Gemini					ChatGPT			
			True (n=192)	False (n=8)	*P* value	Q value	True (n=188)		False (n=12)	*P* value	Q value	True (n=185)		False (n=15)	*P* value	Q value
Word count, median (IQR)		55 (40, 74)	54 (39, 73)	70 (60, 83)	**.**049^a^	0.098	54 (39, 74)		65 (56, 74)	.12^a^	0.175	53 (39, 73)		64 (55, 87)	.04^a^	0.082
**Phase, n (%)**					.60^b^	0.72				.15^b^	1.75				>.99^b^	>.99
	Clinical phase	177 (89)	169 (88)	8 (100)			168 (89)		9 (75)			163 (88)		14 (93)		
	Preclinical phase	23 (12)	23 (12)	0 (0)			20 (11)		3 (25)			22 (12)		1 (6.7)		
**Specialty, n (%)**					>.99^b^	>.99				.49^b^	.498				.15^b^	0.22
	Internal medicine	47 (24)	45 (23)	2 (25)			46 (24)		1 (8.3)			45 (24)		2 (13)		
	Surgery	24 (12)	23 (12)	1 (13)			23 (12)		1 (8.3)			20 (11)		4 (27)		
	Others	129 (65)	124 (65)	5 (63)			119 (63)		10 (83)			120 (65)		9 (60)		

^a^Wilcoxon rank-sum test.

^b^Fisher exact test.

### Accuracy of the Chatbots

**Figure 1:**
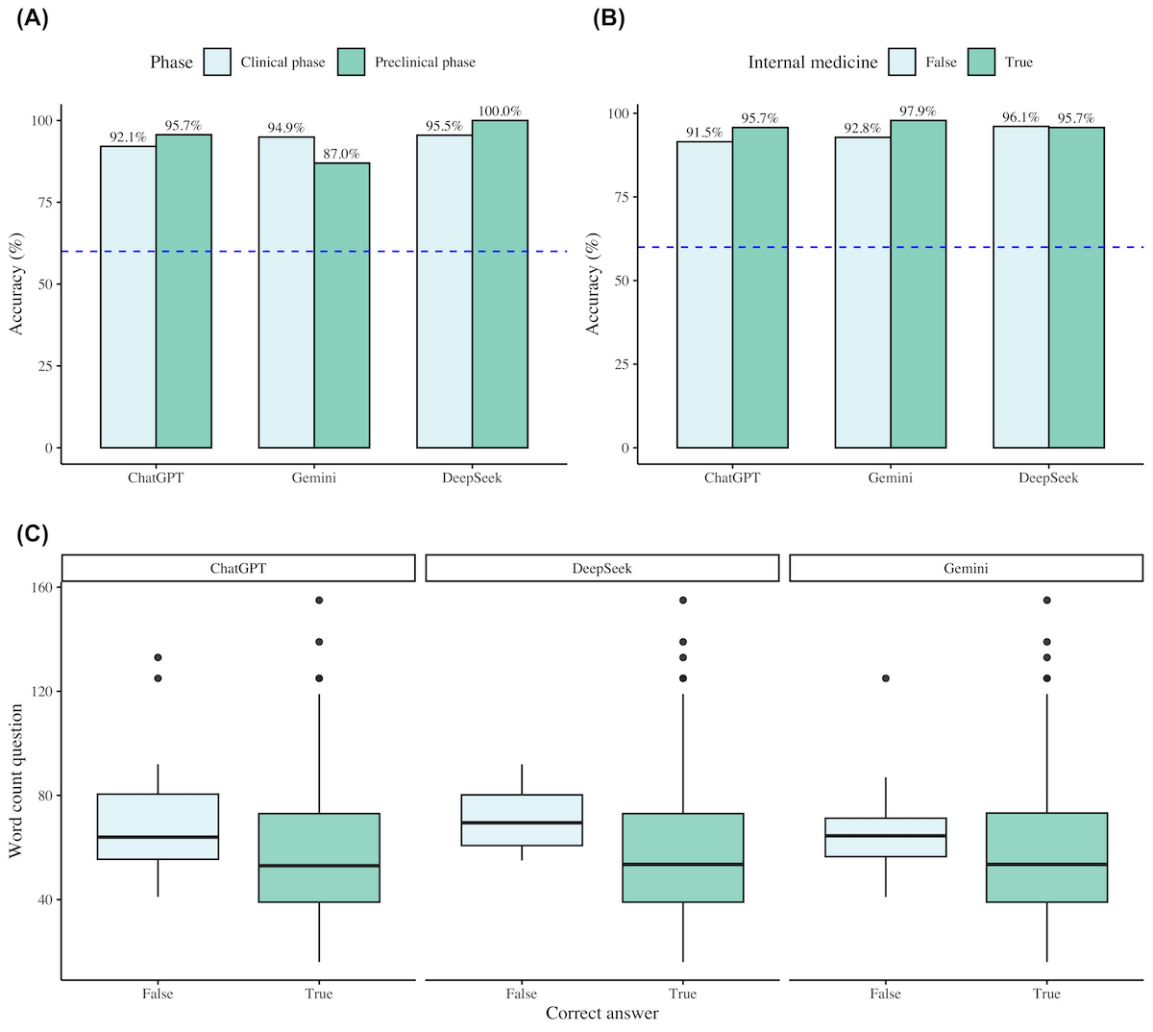
Comparison of ChatGPT, Gemini, and DeepSeek in answering German-language medical multiple-choice questions. (A) Shows the performance categorized by clinical and preclinical phases. (B) Shows the performance on internal medicine questions. The dashed blue line indicates the conventional passing threshold of 60% in the German Progress Test Medicine. (C) Shows a box plot illustrating the relationship between question word count and accuracy.

All chatbot models significantly exceeded the predefined performance threshold of 60% (*P*<.001 for all comparisons). Accuracy was 96% (95% CI 92.9%-100%) for DeepSeek, 94% (95% CI 90.5%-100%) for Gemini, and 92.5% (95% CI 88.7%-100%) for ChatGPT. Accuracy differences among the 3 chatbots were small and not statistically significant, and there was no difference in pairwise comparison after Bonferroni adjustment (Multimedia Appendix 3; Figures 1A and 1B). DeepSeek correctly answered MCQs with a median word count of 54 (IQR 39-73), whereas incorrectly answered questions had a significantly higher median word count of 70 (IQR 60-83; *P*=.049). A similar pattern was also observed for ChatGPT (*P*=.04) but not for Gemini (Figure 1C). No accuracy variations were found across medical specialties or clinical or preclinical categorization (Figures 1A and 1B; Table 1).

### Exploratory Qualitative Assessment of Chatbot Answers

Despite their high accuracy, all 3 chatbots occasionally produced highly plausible but incorrect explanations. In the context of large language models, such errors are commonly referred to as *hallucinations*, meaning the confident generation of factually incorrect or fabricated information that is not supported by the input or external knowledge [30]. For example, in one case, Gemini fabricated an incorrect response label for a multiple-choice answer. In another case, both DeepSeek and ChatGPT proposed a diagnosis that was not among the available options. The clinical scenario described recurrent morning stiffness with enlargement of the distal interphalangeal joints and proximal interphalangeal joints but without redness or swelling. Despite the absence of *arthrosis* from the listed answer choices, both DeepSeek and ChatGPT chose it as the most likely cause. In contrast, the correct answer provided by the questionnaire’s designer was rheumatoid arthritis.

Moreover, some discrepancies among the chatbots appeared to mirror inconsistencies in the medical literature itself [31,32]. For example, in explaining metamizole-induced agranulocytosis, ChatGPT emphasized antibody-mediated granulocyte destruction, whereas DeepSeek and Gemini attributed the condition to direct toxic effects on the bone marrow.

## Discussion

### Principal Findings

In this evaluation of 200 German-language medical MCQs spanning both preclinical and clinical domains, DeepSeek, ChatGPT, and Gemini achieved very high overall accuracies (93%-96%), indicating that an offline‐deployable model, such as DeepSeek, can match leading chatbots in core medical reasoning tasks [10]. Moreover, the mean difficulty index of the PTM 51 was 0.35, corresponding to an average student accuracy of 35% on the same questions [16]. Thus, all 3 chatbots substantially outperformed the comparison group of medical students. Notably, performance for both DeepSeek and ChatGPT declined with increasing question length, underscoring that verbose prompts remain a persistent challenge and echoing previous observations that longer MCQs amplify opportunities for error [18]. Sporadic yet plausible *hallucinations* and *out‐of‐options* answers—well‐documented safety concerns in large language models—were observed across all 3 chatbots [8].

### High Performance of AI-Based Chatbots in MCQs

Compared to earlier ChatGPT versions on the PTM, our results illustrate rapid progress in the previous years [33,34]. Similarly, studies reporting improvements from GPT-3.5 (58%) to GPT-4 (81%) in medical state examinations mirror our findings [5,35]. Moreover, Alfertshofer et al [36] analyzed 1200 medical licensing MCQs and identified question length and language as key determinants of accuracy, mirroring our finding that verbosity negatively impacts performance. Within German-language contexts, Friederichs et al [34] found that ChatGPT answered roughly two-thirds of PTM items correctly and outperformed early-year medical students, consistent with our result that all 3 chatbots surpassed conventional pass thresholds on the PTM 51 item pool. Notably, the accuracies reported in our study exceed those of earlier PTM estimates, underscoring the rapid capability gains since 2023.

Our results also fit into a broader international landscape of benchmarking studies that have consistently reported high but context-dependent performance of generative AI in medical MCQs. DeepSeek-R1, for example, achieved accuracies of 97% on English and Chinese licensing items [1]; 92% to 95% on the Chinese National Medical Licensing Examination, with significant and stable advantages over ChatGPT [2,6]; and more than 90% in oncology [10] and microbiology [11]. In contrast, head-to-head comparisons on the United States Medical Licensing Examination found DeepSeek slightly inferior to ChatGPT-o1 (92% vs 95%) [3], while in ophthalmology board-style examinations, ChatGPT o1 Pro (83.4%) clearly outperformed DeepSeek-R1 (72.5%) [4]. Conversely, in pediatric board preparation questions, DeepSeek-R1 reached 98% accuracy, markedly surpassing ChatGPT-4 (82.7%) [5]. These findings underline that apparent global accuracy masks substantial domain-specific variability, where some models excel in pediatrics or oncology, while others dominate in ophthalmology. Importantly, several studies found minimal overlap in the specific questions missed by different models, suggesting complementary rather than uniform knowledge gaps [5].

Language-specific effects further add nuance to these comparisons. In bilingual ophthalmology MCQs, DeepSeek performed better in Chinese (86.2%) than in English (80.8%), while Gemini and OpenAI models showed weaker robustness across languages [8]. Similar findings in multiyear Chinese National Medical Licensing Examination evaluations confirm DeepSeek’s consistent advantage in Chinese [2,6], supporting the view that training corpus composition can strongly shape performance across languages. This resonates with our observation that even in German-language testing, performance is influenced not only by specialty but also by linguistic and structural features of the questions.

Taken together, previous literature and our findings converge on 3 themes. First, the overall performance of state-of-the-art chatbots on medical MCQs is now consistently at or above medical student thresholds, often exceeding 90%. Second, accuracy varies by language, with models such as DeepSeek particularly advantaged in Chinese settings. Third, accuracy differs by domain, with ophthalmology and pediatrics illustrating opposite outcomes across models. Finally, our demonstration that verbosity predicts chatbot errors echoes the hypothesis by Alfertshofer et al [18] that longer questions amplify opportunities for error [35].

Ophthalmology-specific studies likewise found DeepSeek on par with, or superior to, contemporaneous versions of ChatGPT and Gemini [37,38], although 1 study found it lagged behind ChatGPT on pediatric MCQs, suggesting specialty-dependent effects or influences of question format [39]. Indeed, we observed word count to be a critical determinant of chatbot accuracy on medical MCQs, supporting the hypothesis by Alfertshofer et al [18] that longer questions create more opportunities for error.

### Implications for Safety and Transparency

Consistent with the literature, hallucinations remained a relevant obstacle for the chatbots in addressing medical MCQs [14,40]. Thus, DeepSeek’s *think-aloud* feature, where it exposes intermediate reasoning steps, may help end users detect hallucinations, overgeneralization, and dataset biases, provided these outputs are critically reviewed [12,14,40,41]. In addition, this feature might facilitate the scrutiny of DeepSeek’s novel or out-of-scope reasoning.

However, transparency does not inherently mitigate the risk of generating unsafe content. After all, the literature suggests that DeepSeek produces unsafe responses 10 times more often than ChatGPT [42]. While DeepSeek is released under a permissive license with publicly available model weights, sometimes described as partially open source [10,13,14], the very notion of openness in generative AI remains contested. Many so-called open models are only *open-weight*, sharing parameters but withholding training and fine-tuning data, a practice termed *open-washing* [43]. This highlights that openness is best understood as a graded and multidimensional property rather than a binary state.

Furthermore, DeepSeek’s operation within government‐regulated frameworks [14] highlights the tension between transparency and regulatory compliance. Thus, despite the potential to save time, costs, and personnel resources in clinical and research-related decision-making processes in health care [44], the hope that AI-based chatbots can serve as reliable decision-support tools must be critically questioned at this point in time.

### Future Directions

While DeepSeek, ChatGPT, and Gemini all excel on German-language MCQs, persistent issues, such as hallucinations, bias, and unsafe outputs mentioned in the literature, underscore the need for further refinement of these chatbots. Thus, future research should assess chatbot performance on tasks that demand free-text generation and complex reasoning without predefined answer options in real-world simulated environments. Evaluations should also extend across diverse languages, specialties, and examination formats. In addition, studies need to refine prompt engineering approaches to reduce verbosity-related errors and systematically monitor safety, bias, and regulatory compliance over time while accounting for the threat to reproducibility posed by continuous model updates.

### Limitations

Nevertheless, our reliance on medical MCQs may overestimate real-world clinical utility of such AI-based chatbots because such formats cannot capture the nuance of actual patient-clinician interactions [8]. Furthermore, presenting every item in German limits generalizability to other languages, health care settings, and assessment formats. In the literature on ophthalmology MCQs, for instance, DeepSeek’s accuracy rose from 81% in English to 86% in Chinese, whereas the accuracies of ChatGPT and Gemini fell from 72% to 75% down to 68% to 71%, a pattern attributed to the higher proportion of Chinese tokens in DeepSeek’s training data [38]. This finding challenges the common assumption that multilingual AI models are inherently biased toward the English language [35,45] and highlights how training‐corpus composition can drive language‐specific performance [38]. To address concerns regarding reproducibility, we conducted a stability spot-check on a random subset of 20 questions in September 2025. As the original versions tested between February 21, 2025, and March 4, 2025, were no longer available, we used the most recent publicly accessible default web-based versions at that time (ChatGPT5, DeepSeek [version 3.2], and Gemini 2.5 Flash, all accessed on September 30, 2025). A total of 19 (95%) questions were answered correctly by all models, while 1 (5%) error initially made by ChatGPT (GPT-o3-mini) was now reproduced by DeepSeek (version 3.2). This demonstrates that ongoing model updates can change outcomes, underscoring that our findings are specific to particular model versions and access dates.

### Conclusions

In conclusion, DeepSeek matches Gemini and ChatGPT in accuracy on German-language medical MCQs while offering novel insights and a transparent *thinking‑aloud* glimpse into its reasoning. Yet, recurring hallucinations and documented biases make expert oversight and critical appraisal indispensable. Furthermore, the literature points to potential safety and regulatory concerns that could outweigh DeepSeek’s offline-deployment advantages, such as lower environmental footprint and operational costs. Ultimately, targeted research is needed to delineate DeepSeek’s failure modes, rigorously validate its safety and impartiality, and establish best-practice strategies.

## Appendix

Multimedia Appendix 1 Cleaned data for data analysis.

Multimedia Appendix 2 Code for data analysis.

Multimedia Appendix 3 Accuracy of Gemini, ChatGPT, and DeepSeek in the German Progress Test Medicine.

## Abbreviations

AI: artificial intelligence
MCQ: multiple-choice question
PTM: Progress Test Medicine

## Data Availability

The datasets analyzed during this study are not publicly available due to copyright restrictions but are available from the corresponding author on reasonable request.
