# Supplementary material for: Performance of DeepSeek-R1, ChatGPT (GPT-o3-mini), and Gemini 2.0 Flash on German Medical Multiple-Choice Questions: Comparative Evaluation
Source: JMIR Form Res. 2025 Dec 18;9:e77357. doi: 10.2196/77357 (PMC12757712; doi:10.2196/77357)
Supplement: Multimedia Appendix 2 [file formative_v9i1e77357_app2.pdf]

# Analysis_29.09.25

2025-09-29

```
knitr::opts_chunk$set(echo = TRUE)

######### Importing Libraries #################
library(pwr) # Sample Size Calculation
library(rio) # Data Import
library(tidyverse) # Data Wrangling
```

```
## ── Attaching core tidyverse packages ──────────────────────── tidyverse 2.0.0 ──
## ✔ dplyr     1.1.4     ✔ readr     2.1.5
## ✔ forcats   1.0.0     ✔ stringr   1.5.1
## ✔ ggplot2   3.5.2     ✔ tibble    3.3.0
## ✔ lubridate 1.9.4     ✔ tidyr     1.3.1
## ✔ purrr     1.1.0
## ── Conflicts ────────────────────────────────────────── tidyverse_conflicts() ──
## ✖ dplyr::filter() masks stats::filter()
## ✖ dplyr::lag()    masks stats::lag()
## ℹ Use the conflicted package (<http://conflicted.r-lib.org/>) to force all conflict
s to become errors
```

```
library(gtsummary) # Tables
library(labelled) # Labels for Tables
library(RColorBrewer) # Colors
library(fastDummies) # Creating Dummy-Variables
library(cowplot) # Arranging Plots
```

```
##
## Attaching package: 'cowplot'
##
## The following object is masked from 'package:lubridate':
##
##     stamp
```

```
library(rstatix) # Comparing data
```

```
##
## Attaching package: 'rstatix'
##
## The following object is masked from 'package:stats':
##
##     filter
```

```
library(here) # Data Import
```

```
## here() starts at /Users/annikameyer/Documents/Forschung/DeepSeek/Revision
```

```
######### Importing Data set #################
df <- import("Appendix 1.xlsx") %>%
  mutate(ID = row_number()) %>%
  select(ID, Speciality, Phase, DeepSeek:Wordcount)
```

```
## Required sample size per chatbot: 195
```

# Methods

## Berlin Progress Test

The Progress Test Medicine used is a knowledge test designed at the Charité in Berlin for students of human medicine. It accompanies around 12,000 students from 19 universities in Germany, Austria and Switzerland through their medical studies. The Progress Test Medicine consists of 200 interdisciplinary MCQ at graduate level and is designed to provide students with objective feedback on their personal growth in knowledge over the course of their studies. The Progress Test Medicine was selected as it integrates both preclinical and clinical questions, making it a comprehensive assessment tool. The test results and in particular the knowledge gain per semester have proven to be suitable criteria for predicting academic success with regard to the German state examinations. Each question was categorized by a medical doctor regarding subject area and study phase (clinical, preclinical).

## Data Collection

To address our research question, we evaluated the performance of three chatbots – ChatGPT (GPT-o3-mini), DeepSeek (DeepSeek-R1), and Gemini (Gemini 2.0 Flash) – using 200 multiple-choice questions from the Progress Test Medicine between February 21st and March 4th, 2025. The number of questions was determined based on a sample size calculation for the McNemar test, assuming a Cohen's d of 0.28 and a statistical power of 80 %, yielding a required sample size of 195.

In accordance with the findings of Alfertshofer et al., the word count of each question was subsequently determined (14). The unaltered questions were then presented to each chatbot via their publicly accessible web-based user interfaces. Each question was submitted within a fresh chat session to prevent memory bias or response contamination on the part of the chatbots. On occasions where the chatbot failed to generate a response initially, the query was reinitiated.

## Statistical Analysis

Statistical analyses were performed using 'R'. Sample size calculations were performed with the 'pwr' package. Data wrangling and analysis were carried out using 'rio', 'tidyverse', 'gtsummary', 'rstatix', 'labelled', and 'fastDummies', while data visualization was accomplished using the 'tidyverse', 'RColorBrewer', and 'cowplot' packages. Categorical variables were summarized by absolute and relative frequencies, whereas continuous variables were described using the median and interquartile range. Normality was assessed using the Shapiro-Wilk test. Differences in chatbot performance were evaluated with McNemar's test for paired categorical data and the Fisher's exact test for un-paired categorical data, while the Wilcoxon signed-rank test was applied for continuous data. A *p-value of < 0.05 was considered statistically significant.

```
shapiro.test(df$Wordcount) # Normal Distribution
```

```
##
##  Shapiro-Wilk normality test
##
## data:  df$Wordcount
## W = 0.95732, p-value = 1.025e-05
```

```
table_question <- tbl_summary(df %>%  # Summary Table regarding descriptive statistics
                                select(Wordcount, Speciality, Phase))

crosstable_accuracy_appendix_1 <- tbl_summary(df %>% # Accuracy table for chatbots
                                                select(Gemini, ChatGPT, DeepSeek))
```

```
# Dataset regarding
accuracy_df <- df %>% # Creating the Crosstable as Dataset
  summarize(
    DeepSeek = mean(DeepSeek)*100,
    Gemini = mean(Gemini)*100,
    ChatGPT = mean(ChatGPT)*100
  ) %>%
  pivot_longer(everything(), names_to = "Method", values_to = "Accuracy") %>%
  mutate(Method = factor(Method, levels = c("ChatGPT", "Gemini", "DeepSeek")))

# Accuracy Bar Plot
plot_1 <- ggplot(accuracy_df, aes(x = Method, y = Accuracy, fill = Method)) +
  geom_bar(stat = "identity", width = 0.6, color = "black") +
  geom_hline(yintercept=60, linetype="dashed", color = "blue") +
  labs(title = "",
       x = "",
       y = "Accuracy [%]") +
  theme_minimal()  +
  scale_fill_brewer(palette ="BuGn") +
  theme_classic() +
  theme(text = element_text(family = "Times")) +
  guides(fill="none")

# Dataset for Accuracy per Phase and LLM
accuracy_phase_df <- df %>%
  group_by(Phase) %>%
  summarize(
    DeepSeek = mean(DeepSeek) * 100,
    Gemini   = mean(Gemini)   * 100,
    ChatGPT  = mean(ChatGPT)  * 100
  ) %>%
  pivot_longer(cols = c("DeepSeek", "Gemini", "ChatGPT"),
               names_to = "Method",
               values_to = "Accuracy") %>%
  mutate(Method = factor(Method, levels = c("ChatGPT", "Gemini", "DeepSeek")))

# Grouped Bar Plot
plot_phase <- ggplot(accuracy_phase_df, aes(x = Method, y = Accuracy, fill = Phase)) +
  geom_bar(stat = "identity", position = "dodge", width = 0.7, color = "black") +
  geom_hline(yintercept = 60, linetype = "dashed", color = "blue") +
  labs(title = "",
       x = "",
       y = "Accuracy [%]") +
  theme_minimal() +
  scale_fill_brewer(palette = "BuGn") +
  theme_classic() +
  theme(text = element_text(family = "Times"),
        legend.position = "right")

# Grouped Dataset for Internal Medicine
accuracy_speciality_df <- df %>%
  mutate("Internal\nMedicine" = ifelse(Speciality == "Internal Medicine", T, F)) %>%
  group_by(`Internal\nMedicine`) %>%
  summarize(
    DeepSeek = mean(DeepSeek) * 100,
    Gemini   = mean(Gemini)   * 100,
    ChatGPT  = mean(ChatGPT)  * 100
  ) %>%
  pivot_longer(cols = c("DeepSeek", "Gemini", "ChatGPT"),
               names_to = "Method",
               values_to = "Accuracy") %>%
  mutate(Method = factor(Method, levels = c("ChatGPT", "Gemini", "DeepSeek")))

# Grouped Bar Plot
plot_speciality <- ggplot(accuracy_speciality_df, aes(x = Method, y = Accuracy,
                                                      fill = `Internal\nMedicine`)) +
  geom_bar(stat = "identity", position = "dodge", width = 0.7, color = "black") +
  geom_hline(yintercept = 60, linetype = "dashed", color = "blue") +
  labs(title = "",
       x = "",
       y = "Accuracy [%]") +
  theme_minimal() +
  scale_fill_brewer(palette = "BuGn") +
  theme_classic() +
  theme(text = element_text(family = "Times"),
        legend.position = "right")

# Pivotting long
df_long <- df %>%
  pivot_longer(
    cols = c(Gemini, ChatGPT, DeepSeek),
    names_to = "Chatbots",
    values_to = "Accuracy"
  ) %>%
  mutate(Method = factor(Chatbots, levels = c("ChatGPT", "Gemini", "DeepSeek")))

# Create boxplots for Wordcount by Status, faceted by Platform
boxplot <- ggplot(df_long, aes(x = as.factor(Accuracy),
                               y = Wordcount, fill = as.factor(Accuracy))) +
  geom_boxplot(alpha=0.9) +
  facet_grid(Chatbots~.) +
  labs(
    x = "Correct Answer",
    y = "Wordcount Question",
    title = ""
  ) +
  scale_fill_brewer(palette ="BuGn") +
  theme_classic() +
  theme(text = element_text(family = "Times")) +
  guides(fill="none")

figure_1a <- plot_grid(plot_1, plot_phase, plot_speciality,
                               labels=c("A", "B", "C"), ncol=1)

figure_1 <- cowplot::plot_grid(figure_1a, boxplot,
                               labels = c("", "D"), ncol = 2,
                               rel_widths = c(1.7, 1))


ggsave("Figure_1.tiff", plot = figure_1, width = 8, height = 8, dpi = 600)
```

```
# Creating datasets with certain specialities
df_mutates <- df %>%
  mutate(Speciality = case_when(
    Speciality == "Internal Medicine" ~ "Internal Medicine",
    Speciality == "Surgery" ~ "Surgery",
    TRUE ~ "Others"
  ),
  Speciality = as.factor(Speciality),
  Phase = as.factor(Phase))

# Creating dummy variables
factor_cols <- names(df_mutates)[sapply(df_mutates, is.factor)]

df_dummy <- dummy_cols(df_mutates, select_columns = factor_cols, remove_first_dummy =
FALSE, remove_selected_columns = TRUE)

colnames(df_dummy) <- gsub("^Speciality_|^Phase_", "", colnames(df_dummy))

#df_dummy <- df_dummy %>% select(Wordcount, Clinical, Preclinical, `Internal Medicine
`, Surgery,
                   # Others, DeepSeek, Gemini, ChatGPT)

# Creating the summary tables
DeepSeek <- df_dummy %>%
  select(-c(ID, Gemini, ChatGPT)) %>%
  tbl_summary(by = DeepSeek) %>%
  add_p() %>%
  add_q(method = "bonferroni") %>%
  separate_p_footnotes() %>%
  add_overall()


Gemini <- df_dummy %>% select(-c(ID, DeepSeek, ChatGPT)) %>%
  tbl_summary(by=Gemini) %>%
  add_p()%>%
  add_q(method = "bonferroni")  %>%
  separate_p_footnotes()

ChatGPT <- df_dummy %>% select(-c(ID, Gemini, DeepSeek)) %>%
  tbl_summary(by=ChatGPT) %>%
  add_p() %>%
  add_q(method = "bonferroni") %>%
  separate_p_footnotes()

# Creating overview table
tbl_all <- tbl_merge(
  tbls = list(DeepSeek, Gemini, ChatGPT),
  tab_spanner = c("**DeepSeek**", "**Gemini**", "**ChatGPT**"))
```

```
############### Unterscheiden sich die Chatbots statistisch
df_chatbot <- df %>%
  pivot_longer(
    cols = c("Gemini", "ChatGPT", "DeepSeek"),
    names_to = "Chatbot",
    values_to = "Response"
  )

xtabs(~Response + Chatbot, df_chatbot)
```

```
##         Chatbot
## Response ChatGPT DeepSeek Gemini
##    FALSE      15        8     12
##    TRUE      185      192    188
```

```
cochrane <- cochran_qtest(df_chatbot, Response ~ Chatbot|ID)

mcnemar_appendix_2 <- pairwise_mcnemar_test(df_chatbot, p.adjust.method = "bonferron
i",
                                            Response ~ Chatbot|ID)
```

# Results

## Questions

A total of 200 questions were used to evaluate the three chatbots, with an average length of 55 (40, 74) words. The majority 89% (177 / 200) focused on clinical knowledge, with nearly one-quarter specifically addressing internal medicine (Table 1).

```
tbl_all
```

| | DeepSeek | | | | | Gemini | | | | |
|---|---|---|---|---|---|---|---|---|---|---|
| **Characteristic** | **Overall** N = 200[1] | **FALSE** N = 8[1] | **TRUE** N = 192[1] | **p-value** | **q-value**[2] | **FALSE** N = 12[1] | **TRUE** N = 188[1] | **p-value** | **q-value**[2] | **FALSE** N = 15[1] |
| Wordcount | 55 (40, 74) | 70 (60, 83) | 54 (39, 73) | 0.049[3] | 0.3 | 65 (56, 74) | 54 (39, 74) | 0.11[3] | 0.7 | 64 (55, 87) |
| Internal Medicine | 47 (24%) | 2 (25%) | 45 (23%) | >0.9[4] | >0.9 | 1 (8.3%) | 46 (24%) | 0.3[4] | >0.9 | 2 (13%) |
| Others | 129 (65%) | 5 (63%) | 124 (65%) | >0.9[4] | >0.9 | 10 (83%) | 119 (63%) | 0.2[4] | >0.9 | 9 (60%) |
| Surgery | 24 (12%) | 1 (13%) | 23 (12%) | >0.9[4] | >0.9 | 1 (8.3%) | 23 (12%) | >0.9[4] | >0.9 | 4 (27%) |
| Clinical | 177 (89%) | 8 (100%) | 169 (88%) | 0.6[4] | >0.9 | 9 (75%) | 168 (89%) | 0.15[4] | 0.9 | 14 (93%) |
| Preclinical | 23 (12%) | 0 (0%) | 23 (12%) | 0.6[4] | >0.9 | 3 (25%) | 20 (11%) | 0.15[4] | 0.9 | 1 (6.7%) |

[1] Median (Q1, Q3); n (%)

[2] Bonferroni correction for multiple testing

[3] Wilcoxon rank sum test

[4] Fisher's exact test

[5] Pearson's Chi-squared test

## Accuracy of the Chatbots

DeepSeek achieved the highest accuracy at 96% (192 / 200, followed by Gemini at 94% (188 / 200 and ChatGPT at 93% (185 / 200. All three chatbots exceeded the conventional passing threshold of 60%. Despite DeepSeek's numerically superior accuracy, no statistically significant difference in overall accuracy was detected across the three chatbots (p = 0.099), nor in pairwise comparisons (p > 0.05) (Figure 1A, Appendix 1). Further analysis showed that question wordcount was significantly different in regard to accuracy for DeepSeek p=0.049 and ChatGPT p=0.041, whereas no significant difference was found for Gemini. Additionally, neither specialty area nor classification as clinical versus preclinical was associated with accuracy (p > 0.05) (Figure 1B-D, Appendix 2).

```
figure_1
```

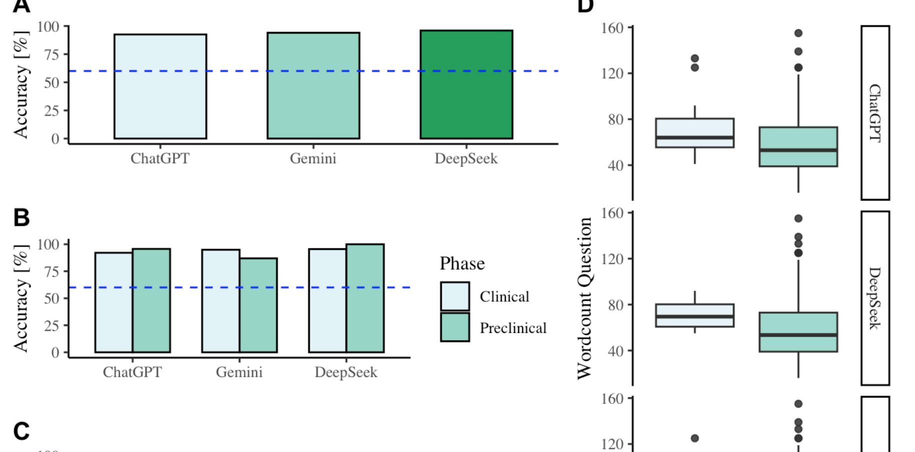


## Exploratory qualitative assessment of chatbot errors

Despite their high accuracy, all three chatbots occasionally produced highly plausible but incorrect explanations. Although rare, technical mistakes did occur. Gemini mislabelled the correct response letter in one instance.

Notably, DeepSeek and ChatGPT sometimes proposed diagnoses that were more suitable than any of the multiple-choice options provided. In one scenario, which described recurrent morning stiffness with bony enlargement of DIP and PIP joints but no signs of redness or swelling, both DeepSeek and ChatGPT recommended osteoarthrosis—a diagnosis not listed among the answer choices—as the most likely cause.

Discrepancies among the chatbots sometimes reflected inconsistent information in the medical literature itself. For example, in explaining metamizole-induced agranulocytosis, ChatGPT emphasized antibody-mediated granulocyte destruction, whereas DeepSeek and Gemini attributed the condition to direct toxic effects on the bone marrow.

```
library(broom)
df_summary <- df %>%
  summarise(
    DeepSeek_correct = sum(DeepSeek, na.rm = TRUE),
    Gemini_correct   = sum(Gemini, na.rm = TRUE),
    ChatGPT_correct  = sum(ChatGPT, na.rm = TRUE),
    n = n()
  )

df_summary
```

```
##   DeepSeek_correct Gemini_correct ChatGPT_correct   n
## 1              192            188             185 200
```

```
results <- bind_rows(
  tidy(binom.test(df_summary$DeepSeek_correct, df_summary$n, p = 0.60, alternative = "
greater")) %>% mutate(Model = "DeepSeek"),
  tidy(binom.test(df_summary$Gemini_correct, df_summary$n, p = 0.60, alternative = "gr
eater")) %>% mutate(Model = "Gemini"),
  tidy(binom.test(df_summary$ChatGPT_correct, df_summary$n, p = 0.60, alternative = "g
reater")) %>% mutate(Model = "ChatGPT")
) %>%
  select(Model, estimate, conf.low, conf.high, p.value)

results
```

```
## # A tibble: 3 × 5
##   Model    estimate conf.low conf.high  p.value
##   <chr>       <dbl>    <dbl>     <dbl>    <dbl>
## 1 DeepSeek    0.96     0.929         1 9.78e-33
## 2 Gemini      0.94     0.905         1 2.22e-28
## 3 ChatGPT     0.925    0.887         1 1.62e-25
```
