# Supplementary material for: Performance of DeepSeek-R1, ChatGPT (GPT-o3-mini), and Gemini 2.0 Flash on German Medical Multiple-Choice Questions: Comparative Evaluation
Source: JMIR Form Res. 2025 Dec 18;9:e77357. doi: 10.2196/77357 (PMC12757712; doi:10.2196/77357)
Supplement: Multimedia Appendix 3 [file formative_v9i1e77357_app3.docx]

**Appendix 1: Accuracy of Gemini, ChatGPT and DeepSeek in the Progress Test Medicine.**

| **Characteristic** | **N = 200***^1^* |
| --- | --- |
| Gemini | 188 (94%) |
| ChatGPT | 185 (93%) |
| DeepSeek | 192 (96%) |
| *^1^* n (%) |  |
